# Diagnostic and prognostic significance of keloid-like collagen remodeling patterns in the extracellular matrix of colorectal cancer

**DOI:** 10.3389/pore.2024.1611789

**Published:** 2024-06-06

**Authors:** Nauryzbay M. Imanbayev, Yerbolat M. Iztleuov, Yevgeniy K. Kamyshanskiy, Aigul V. Zhumasheva

**Affiliations:** ^1^ Department of Oncology, West Kazakhstan Marat Ospanov Medical University, Aktobe, Kazakhstan; ^2^ Department of Radiology, West Kazakhstan Marat Ospanov Medical University, Aktobe, Kazakhstan; ^3^ Department of Pathology, Karaganda Medical University, Karaganda, Kazakhstan; ^4^ Department of Pathomorphology, Medical Centre of West Kazakhstan Marat Ospanov Medical University, Aktobe, Kazakhstan

**Keywords:** colorectal cancer, intermediate desmoplastic reaction, keloid-like collagen, desmoplastic reaction, extracellular matrix of colorectal cancer

## Abstract

**Background:**

The desmoplastic reaction is considered a promising prognostic parameter for colorectal cancer. However, intermediate desmoplastic reaction is characterized by sizeable stromal heterogeneity, including both small amounts of keloid-like collagen (KC) in the fibrotic stroma and thick tufts of KC circumferentially surrounding cancer nests and occupying most of the fields of view. The present study aimed to evaluate the diagnostic and prognostic significance of KC histophenotyping with a quantitative visual assessment of its presence in the stroma of the invasive margin of TNM (The “tumor-node-metastasis” classification) stage II/III colorectal cancer (CRC).

**Methods and results:**

175 resected tumors from patients with TNM stage II/III CRC were examined. Keloid-like collagen was assessed according to Ueno H. criteria. KC was assessed at the primary tumor invasive margin using Hematoxylin & Eosin and Masson’s trichrome staining. The cut-off point for KC was examined using “the best cutoff approach by log-rank test.” Using a cutoff point of 30%, we histologically divided fibrous stroma in the invasive area into two groups: “type A”—KC ≤ 0.3 and “type B”—KC>0.3. Type A stroma was observed in 48% of patients, type B—in 52%. The association between collagen amount and 5-year recurrence-free survival (5-RFS) was assessed using Cox regression analysis. Kaplan-Meier analysis and log-rank tests were used to assess the significance of survival analysis. Analysis of categorical variables showed that increased KC in CRC stroma predicted adverse outcomes for 5-RFS (hazard ratio [HR] = 3.143, 95%, confidence interval [CI] = 1.643–6.012, *p* = 0.001). Moreover, in Kaplan-Meier analysis, the log-rank test showed that type B exhibited worse 5-RFS than type A (*p* = 0.000).

**Conclusion:**

KC is an independent predictor of 5-year overall and RFS in patients with TNM stage II/III CRC treated with surgery, with worse survival rates when the amount of KC increases by >30%.

## Introduction

Colorectal cancer (CRC) is a disease with high morbidity and mortality, ranking third in prevalence worldwide [1]. One basis for making clinical decisions about the treatment of cancer patients, including patients with CRC, is the TNM classification [2]. However, patients with the same stage of CRC, according to the TNM classification, have different clinical outcomes. In this regard, searching for additional prognostic markers to stratify patients and improve clinical decisions is exceptionally relevant. Histological examination is highly reliable and inexpensive, so identifying additional histological criteria is a promising strategy.

Traditionally, the leading place in histological studies was occupied by the epithelial component, but modern research paradigms have gradually shifted from the tumor epithelium to the stroma [3–5]. Even Masson P. drew attention to proliferating fibroblasts among the inflammatory infiltrate of the tumor and described the reactions of the tumor stroma depending on the time of their appearance [6]. The prognostic value of the stroma of the invasive tumor margin was demonstrated by Ueno H [5, 7, 8]. The desmoplastic reaction is considered a promising prognostic parameter for colorectal cancer. However, the intermediate desmoplastic reaction is characterized by sizeable stromal heterogeneity, including both small amounts of keloid-like collagen in the fibrotic stroma and thick tufts of colloid-like collagen circumferentially surrounding cancer nests and occupying most of the visual fields.

Recent studies have shown that tumors can directly exploit extracellular matrix remodeling to create a microenvironment that promotes tumorigenesis and metastasis [9, 10]. The dominant component of the extracellular matrix is collagen, and its structure is increasingly recognized as a reliable biomarker for establishing the prognosis of various types of tumors, such as prostate cancer and gastric cancer [11, 12]. In addition, previous studies have shown that different histopatterns of extracellular collagen matrix is associated with different therapy response in colorectal and breast cancer [13–15]. Collagen I fibers influence the movement of macromolecules in the tumor extracellular matrix [16], and the structural characteristics of these fibers can promote or hinder cancer cell migration [17]. Collagen changes in the tumor microenvironment correlate with cancer dissemination and prognosis. Collagen regulates tumor-associated immune infiltration and is required for tumor angiogenesis, histological features of the Collagen I matrix can lead to significantly enhanced tumor metastatic potential [18, 19]. Moreover, radial alignment of collagen at the tumor-stroma interface increases the invasiveness of cancer cells [20, 21]. Histologically, abnormal collagen remodeling mainly results in excessive deposition and changes in the proportions and localization of collagen [22–24]. These morphological changes may reflect important tumor characteristics that influence patient prognosis.

Pathologists can identify stromal components, but their description and quantification is not routine and stromal factors are not always considered when making treatment decisions. This leads to different approaches in understanding the nature of individual tumors between the laboratory and the clinic. Additional evaluation of sections stained with hematoxylin and eosin does not always allow for the best determination of the various elements of the extracellular matrix. The purpose of the work was to evaluate the diagnostic and prognostic significance of histophenotyping of keloid-like collagen with a quantitative visual assessment of its presence in the stroma of the invasive margin of TNM stage II-III colorectal cancer.

## Materials and methods

### Tissue samples and study cohort selection

This monocentric retrospective cohort study consecutively included resected colorectal cancer specimens with keloid-like collagen at the invasive tumor margin from all patients who underwent complete oncologic resection for TNM stage II or III colon adenocarcinoma in Aktobe (Kazakhstan) during the period since January 2001 until December 2019 All patients were observed for 5 years from the date of surgical resection.

Exclusion criteria: 1) histological diagnosis other than adenocarcinoma; 2) tumor-stroma ratio less than 50%; 3) desmoplastic reaction of the invasive tumor margin, classified as “immature,” myxoid stroma according to Ueno H. criteria [5]; 4) radiation therapy before surgery;

5) Patient death within 30 days after surgery; 6) history of malignancy within 5 years before the diagnosis of colorectal cancer (except basal cell carcinoma or cervical cancer *in situ*); 7) patient refusal of treatment, highly severe clinical condition of the patient, or old age (>90 years). All tissues selected for the study were re-evaluated independently by two pathologists without their knowledge of the clinical information. All samples were anonymized before the start of the study.

Keloid-like collagen was assessed at the invasive margin of the primary tumor using hematoxylin & eosin and Masson’s trichrome staining.

**FIGURE 1:**
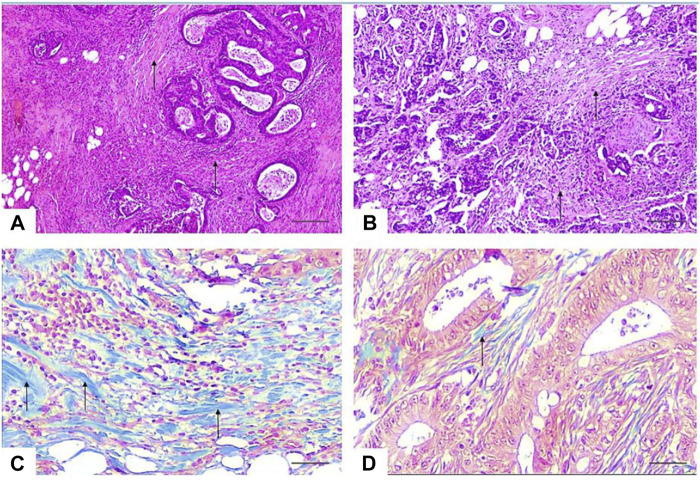
Photomicrographs of representative sections of the invasive tumor margin. **(A, B)**—the stroma-to-tumor ratio is more than 50%, the stroma is dense with multiple bundles of keloid-like collagen (arrows) with bright eosinophilic hyalinization around tumor nests and focal infiltration by immune cells. HE × 40. Scale bar, 500 μm **(C)**—stromal keloid-like collagen histopattern type B—thick tufts of hypocellular keloid-like collagen (arrows) of a homogeneous blue color, occupy more than a third of the stroma of the invasive margin of the tumor. Masson’s trichrome × 400. Scale bar, 50 μm. **(D)**—Stromal mature collagen histopattern type A—thin mature collagen fibers (arrows) and single keloid-like collagen fibers. Masson’s trichrome 400. Scale bar, 50 μm.

In all our cases, two pathologists (Y.K.K. and A.V.Z.) with more than 10 years of experience identified the most invasive part of the removed tumor (“the invasion front”), defined as the area with the deepest tumor infiltration or the area where tumor tissue meets non-tumor tissue. On a microslide of the most invasive edge of the tumor 10 fields of microscopic view with ×40 objective magnification were selected, and then the relative amount of keloid-like collagen was calculated for each case. The cut-off point for keloid-like collagen was examined using “the best cut-off approach by log-rank test” and 30% was determined as the cut-off point. All cases, based on the ratio of colloid-like collagen in the stroma of the invasive edge of the tumor, were divided into groups “type A” (Keloid-like Collagen ≤ 0.3) and “type B” (Keloid-like Collagen > 0.3) (Table 1; Figure 1).

**TABLE 1:** Clinicopathological characteristics of the investigated groups.

Parameters	Categories	Total *n* = 175	Тype А *n* = 84	Тype В *n* = 91	*p*-value
Age (years)	ME ± SD	61.8 ± 9.9	60.9 ± 10.6	62.7 ± 9.4	0.332
	Range	[38; 89]	[38; 81]	[40; 89]	
	≤65 years	107 (61.1)	54 (64.3)	53 (58.2)	
	>65 years	68 (38.9)	30 (35.7)	38 (41.8)	
Gender, n (%)	Male	85 (48.6)	40 (47.6)	45 (49.5)	0.809
	Female	90 (51.4)	44 (52.4)	46 (50.5)	
Race, n (%)	White	43 (24.6)	26 (31.0)	17 (18.7)	0.060
	Asian	132 (75.4)	58 (69.0)	74 (81.3)	
	Other	—	—	—	
CCI Score	≤3	109 (62.3)	48 (57.1)	61 (67.0)	0.178
	>3	66 (37.7)	36 (42.9)	30 (33.0)	
Family history of cancer, n (%)	No	148 (84.6)	71 (84.5)	77 (84.6)	0.987
	Yes	27 (15.4)	13 (15.5)	14 (15.4)	
Date of diagnosis	2001–2005	20 (11.4)	8 (9.5)	12 (12.4)	0.553
	2006–2010	35 (20.0)	14 (16.7)	21 (21.6)	
	2011–2015	57 (32.6)	30 (35.7)	27 (27.8)	
	2016–2019	63 (36.0)	32 (38.1)	31 (31.9)	
Tumor site, n (%)	Right	68 (38.9)	30 (35.7)	38 (41.8)	0.413
	Left	107 (61.1)	54 (64.3)	53 (58.2)	
T-stage, n (%)	T2	127 (72.6)	68 (81.0)	59 (64.8)	**0.017**
	T3	48 (27.4)	16 (19.0)	32 (35.2)	
Tumor differentiation, n (%)	G1	2 (1.1)	1 (1.2)	1 (1.1)	**0.020**
	G2	134 (76.6)	72 (85.7)	62 (68.1)	
	G3	39 (22.3)	11 (13.1)	28 (30.8)	
Tumor budding	BD1	69 (39.4)	39 (46.4)	30 (33.0)	**0.036**
	BD2	67 (38.3)	33 (39.3)	34 (37.4)	
	BD3	39 (22.2)	12 (14.3)	27 (29.6)	
Lymphatic invasion	Negative	132 (75.4)	70 (83.3)	62 (68.1)	**0.020**
	Positive	43 (24.6)	14 (16.7)	29 (31.9)	
Venous invasion	Negative	130 (74.3)	69 (82.1)	61 (67.0)	**0.023**
	Positive	45 (25.7)	15 (17.9)	30 (33.0)	
Perineural invasion, n (%)	No	159 (90.9)	75 (89.3)	84 (92.3)	0.489
	Yes	16 (9.1)	9 (10.7)	7 (7.7)	
KRAS status, n (%)	Mutant	100 (57.1)	47 (56.0)	53 (58.2)	0.760
	Wild-type	75 (42.9)	37 (44.0)	38 (41.8)	
Chemotherapy in postoperative period	No	43 (24.6)	17 (20.2)	26 (26.8)	0.352
	Yes	51 (29.2)	24 (28.6)	27 (27.8)	
	No data	81 (46.3)	43 (51.2)	38 (39.2)	

Type А—Keloid-like Collagen ≤ 0.3, Type В—Keloid-like Collagen > 0.3; CCI, Charlson Comorbidity Index; *p*- value—A chi-square test was performed to compare categorical data. Analysis of variance was performed to compare continuous variables, **bold** indicates values with a significant difference, *р* < 0.05.

### Clinical data

Clinical data were collected using software in an integrated health information system from patient medical records. These data included the sex and age of the patients, comorbidities, family history of colorectal cancer, date of surgery, data on concomitant diseases and comorbidities before surgery, localization of the tumor in the right or left side of the colon, occurrence of recurrence, date of its discovery and treatment provided.

### Endpoints and definitions

The primary endpoint of this study was recurrence-free survival (RFS). RFS was defined as the time in months between the date of surgery and the date of cancer recurrence (defined as the first date of radiographic or histological diagnosis of local tumor recurrence or metastasis of colon cancer) or the date of last follow-up (with a maximum period of 5 years). Standard examinations were performed following national cancer surveillance guidelines for colon cancer after curative resection for 5 years after surgery [25]. Patients dying without cancer recurrence were censored at the date of death.

Overall survival was defined as the duration from surgery to death or last follow-up. Patients alive at the last follow-up were recorded as censored events.

Surgical resection was defined as complete single-stage removal of all gross tumors with negative surgical margins on microscopic examination. Distant metastases were determined by preoperative abdominal ultrasound or computed tomography, chest radiography or MRI (Magnetic Resonance Imaging), and intraoperative examination.

The “tumor-node-metastasis” (TNM) classification was used to determine the primary tumor stage according to the edition in effect at the time of cancer diagnosis.

Tumor budding was defined as an isolated cancer cell or cluster comprising <5 cells at the invasive front and classified into grades BD1, BD2, and BD3 according to international criteria [26, 27].

Vascular invasion was defined as the presence of tumor cells in the muscular layer of blood vessels or invasion of the muscular vascular endothelium; lymphatic invasion was defined as the presence of nests of tumor cells in the lymphatic cavity [28].

Perineural invasion was defined as the presence of tumor cells in three layers of the nerve sheath or in close proximity to the nerve, affecting at least 33% of its entire circumference [29–31].

### Tumor marker KRAS

Tissue blocks from all study participants were obtained from the initial surgical resection and assessed for KRAS mutational status. Sequencing of KRAS, representative tumor portions were marked histologically, and the corresponding areas on unstained tissue slides were then subjected to manual microdissection. The dissected tissues were collected into microtubes containing lysis buffer and proteinase K and incubated at 55°C for up to 2 days. KRAS mutations were determined by standard PCR followed by Sanger sequencing of exons 2 and 3 of KRAS.

### Histological examination

Before the histological examination, tissue samples were fixed in 10% formaldehyde at 4°C for 24 h, washed with tap water, and dehydrated using a series of alcohols of increasing concentration (70%, 90%, 95%, 100%). The tissue samples were then immersed in xylene and embedded in paraffin blocks. Tissue sections 3 μm thick were cut using a microtome and placed on a glass slide. The slides were then deparaffinized and stained.

Hematoxylin and eosin staining procedure. Tissue sections were immersed in Mayer’s hematoxylin for 15 min and then washed with water for 5 min. After this, the sections were subjected to 1-min eosin staining.

From the tissue block, we carefully selected plain HE-stained sections that showed the representative invasive part of the primary tumor.

Masson’s trichrome staining procedure. For staining with Masson’s trichrome, a commercial kit [Trichrome dye (Masson) Bio-Optica (Italy)] was used according to the standard protocol. Collagen fibers were defined as dark-blue fibers with black nuclei.

### Morphological criteria

Morphological variables were obtained as a result of repeated review of histological specimens of the tumor (surgical material) and also collected from pathologists’ reports, including tumor differentiation, tumor budding, lymphatic, venous, and perineural invasion.

### Ethics statement

Due to the retrospective nature of this study, ethical review and approval was not required in accordance with the local legislation and institutional requirements.

### Statistical analysis

Statistical analysis was performed using Statistica 10.0 and IBM SPSS Statistic 25.0 software (v.25.00, IBM Statistics, Armonk/NY, United States). No formal sample size calculation was performed. All eligible patients were included in the analysis. Descriptive statistics were used to present the data. Qualitative and quantitative variables were compared using the χ^2^ or Student’s t-test, respectively (Mann-Whitney or Kruskal-Wallis test when the reliability conditions of Student’s t-test and χ^2^ test were not met). A *p*-value <0.05 was considered statistically significant. The Kaplan-Meier method was used to calculate overall (OS) and relapse-free (RFS) survival. Univariate and multivariate analyses using the Cox proportional hazards regression model were performed to calculate HRs and 95% CI. The log-rank test, Kaplan-Meier method, and Cox regression (univariate) were used to estimate the cutoff value.

## Results

### Study population

The clinicopathological characteristics of the study groups of patients are presented in Table 1.

Patients were divided into groups “type A” (Keloid-like Collagen ≤0.3) and “type B” (Keloid-like Collagen > 0.3). Based on the cut-off point, 84 (48%) patients were identified as cases with a reduced amount of keloid-like collagen (type A), and 91 (52%) patients were identified as cases with an increased amount of keloid-like collagen (type B). Representative examples of images with stroma type A and type B histophenotypes are shown in Figure 1.

### Survival analyses of overall survival and recurrence-free survival

**FIGURE 2:**
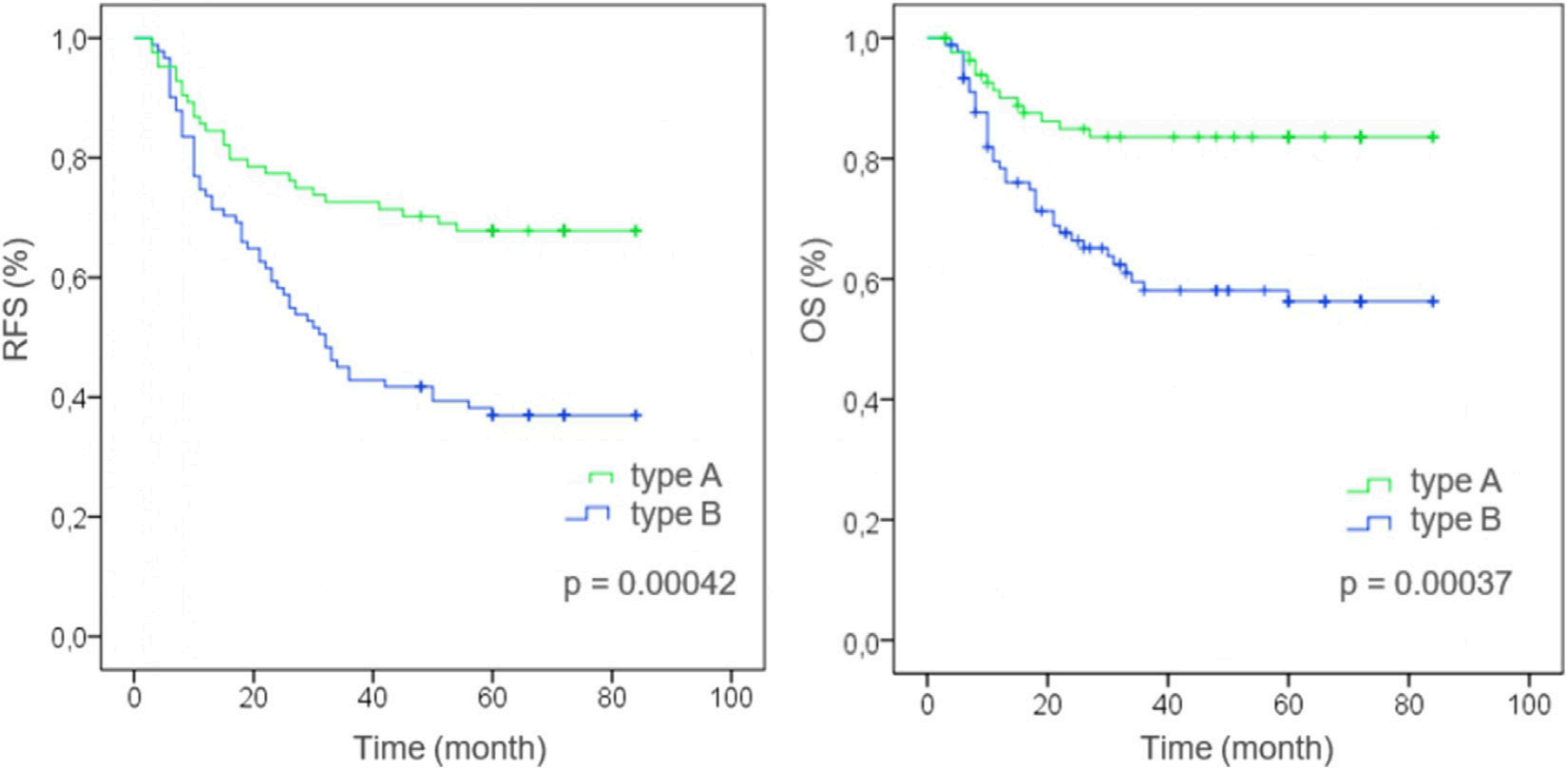
Kaplan-Meier curve features in the investigated groups [stratified by histophenotype of stromal collagen (Type A or Type B) in the colorectal cancer]. RFS—Recurrence-free survival of patients, OS—Overall survival of patients, Type A—Keloid-like Collagen less than 30% of stromal collagen fibers in the invasive tumor front (≤0.3), Type B—Keloid-like Collagen more than 30% of stromal collagen fibers in the invasive tumor front (>0.3).

The results of the Kaplan-Meier analysis of survival curves are presented in Figure 2.

In the group with the structural pattern of mature thin-structured collagen stroma type A, the following survival rates (RFS) were determined: on the 1st year it was 84.5%, on the 3rd year—72.6%, on the 5th year—67.9%; OS in the 1st year was 90.5%, in the 3rd year—84.5%, in the 5th year—84.5%.

In the group with the structural pattern of intermediate keloid-like collagen stroma type B, the following survival rates (RFS) were determined: on the 1st year it was 74.7%, on the 3rd year—48.4%, on the 5th year—37.4%; OS in the 1st year was 80.2%, in the 3rd year—63.7%, in the 5th year—59.4%.

### Univariate and multivariate analysis of prognostic factors

Univariate Cox regression was performed to evaluate the prognostic significance of all clinicopathological data and keloid-like collagen assessed on all slides containing the invasive tumor margin (Table 2).

**TABLE 2:** Univariate and multivariate analyses of RFS using the Cox proportional-hazards regression model in the investigated groups.

		Univariate analysis		Multivariate analysis	
		HR (95% CI)	*p*-value	HR (95% CI)	*p*-value
Age (years)	≤65 years				
	>65 years	1.140 (0.620–2.095)	0.673	—	—
Gender, n (%)	Male				
	Female	1.204 (0.539–2.446)	0.607	—	—
Race, n (%)	White				
	Asian	0.845 (0.425–1.683)	0.633	—	—
CCI Score	≤3				
	>3	2.127 (0.962–4.701)	0.062	—	—
Family history of cancer, n (%)	No				
	Yes	1.212 (0.504–2.916)	0.668	—	—
Tumor site, n (%)	Right				
	Left	0.406 (0.213–1.087)	0.068	—	—
T-stage, n (%)	T2				
	T3	2.345 (1.054–5.215)	**0.037**	1.654 (0.695–3.939)	0.256
Tumor differentiation, n (%)	G1				
	G2	0.375 (0.022–6.484)	0.500	—	—
	G3	1.417 (0.645–3.114)	0.386	—	—
Tumor budding	BD1				
	BD2	2.326 (0.959–5.641)	0.062	—	—
	BD3	1.355 (0.576–3.190)	0.487	—	—
Lymphatic invasion	Negative				
	Positive	2.533 (1.238–5.183)	**0.011**	1.420 (0.495–4.070)	0.514
Venous invasion	Negative				
	Positive	3.509 (1.682–7.323)	**0.001**	1.696 (0.624–4.609)	0.301
Perineural invasion, n (%)	No				
	Yes	2.592 (0.861–7.803)	0.090	—	—
KRAS status, n (%)	Mutant				
	Wild-type	1.046 (0.575–1.902)	0.884	—	—
Keloid-like Collagen	Type А				
	Type В	3.539 (1.895–6.609)	**0.000**	3.143 (1.643–6.012)	**0.001**

Type А—Keloid-like Collagen ≤0.3, Type В—Keloid-like Collagen >0.3; CCI, Charlson Comorbidity Index; *p*-value—**bold** indicates values with a significant difference, *р* < 0.05.

Univariate analysis showed that among the prespecified clinicopathological prognostic factors assessed in this study, the T-stage had a significant impact on RFS: in patients with the T3 stage, the hazard ratio was 2.345 (95% CI: 1.054–5.215; *p* = 0.037) compared to patients at stage T2. Lymphatic invasion [HR, 2.533 (1.238–5.183), *p* = 0.011] and venous invasion [HR, 3.509 (1.682–7.323), *p* = 0.001] were also significant prognostic factors for RFS. Patients with stromal histophenotype Type B (Keloid-like Collagen > 0.3) had a significantly higher risk of relapse [HR, 3.539 (1.895–6.609), *p* = 0.001] than patients with stromal histophenotype Type A. In a univariate analysis, it was found that several factors, such as age, sex, race, family history of cancer, and tumor differentiation grade, did not have a statistically significant effect on RFS in patients with stage II-III colorectal cancer.

After univariate analysis, significant factors were further assessed using a multivariate Cox regression model to determine their independent prognostic value for RFS. After adjustment for other covariates, T stage [HR: 1.654, 95% CI: (0.695–3.939); *p* = 0.256], lymphatic invasion (HR: 1.420, 95% CI: 0.495–4.070; *p* = 0.514) and venous invasion (HR: 1.696, 95% CI: 0.624–4.609; *p* = 0.301) were not predictive of poor outcome, in contrast to the structural pattern of keloid-like collagen. Multivariate analysis showed that stromal histophenotype Type B (Keloid-like Collagen >0.3) was an independent factor significantly associated with worse RFS [HR, 3.143 (1.643–6.012), *p* = 0.001] in multivariate analysis (Table 2).

## Discussion

In this study, we examined the diagnostic and prognostic significance of structural histochemical patterns of keloid-like collagen in the stroma of colorectal cancer. We analyzed the impact of clinical and histopathological prognostic factors on metastasis and survival of patients with colorectal cancer. The histopathological pattern of colon cancer between the left and right parts of the large intestine did not show significant differences, therefore, in this study, the main results of the comparative clinical and morphological assessment are presented as general data from all parts of the large intestine with a relatively uniform number of cases (Table 1).

First, our results showed that increased content of keloid-like collagen in the stroma of the invasive tumor margin correlates with the presence of aggressive features, such as tumor differentiation (G3) (*p* = 0.020), tumor budding (BD3) (*p* = 0.036), lymphatic invasion (*p* = 0.020) and venous invasion (*p* = 0.023).

Second, we found that the categorization of keloid-like collagen at the invasive margin of colorectal cancer stratifies the risk of recurrence in this group of patients. Multivariate models analysis showed that the increase in keloid-like collagen in the stroma is an independent factor of postoperative relapse in patients with stage II-III colon cancer. In particular, distant metastases and postoperative relapses within 5 years after surgery in the group with a keloid-like collagen content of more than 30% were detected in 39.5% of patients, and in the group with a keloid-like collagen content of less than 30% in 15.5% of patients. These results are consistent with scientific research [32–34] and suggest a close relationship between keloid-like collagen and the metastatic behavior of cancer cells.

The mechanism leading to a worsening prognosis in patients with increased keloid-like collagen in the stroma is not yet fully understood. As colorectal cancer progresses the increase in the number of thick collagen fibers may initially be associated with a primary fibroproliferative reaction and, in later stages, with abnormal collagen production and defective maturation, which may promote tumor growth [35]. We believe that the tumor stroma is initially capable of exerting an inhibitory effect on malignant cells.

With continued growth, the tumor can exploit its stroma, for example, by changing its composition, which is morphologically manifested by changes in the quantitative and qualitative characteristics of collagen, in order to stimulate tumor growth and metastasis. This process occurs parallel to tumor progression and is characterized by a complex bidirectional communication between the tumor and its stroma [36–38]. The different rates of progression, relapse, and continued growth suggest that the in some cases tumor progression in the group with increased amount of keloid-like collagen stroma may be caused by primary stromal changes causing *de novo* tumor development. However, this assumption is speculative and requires further research.

Third, Kaplan-Meier analysis revealed that patients with more than 30% keloid-like collagen in the stroma of the invasive tumor margin had a significantly worse survival rate than patients with less than 30% keloid-like collagen (*p* < 0.01). Our results indicate that the predictive power of keloid-like collagen categorization at the invasive margin of colorectal cancer may be independent of the anatomical TNM grade of the disease. These results suggest that stromal classification may provide reliable prognostic features and improve TNM classification for colorectal cancer. In addition, integrating histophenotype into multivariate analysis showed improved predictive capabilities, indicating that this histophenotype may be a potential addition to an outcome prediction system while simultaneously facilitating improved risk stratification for adverse outcomes. The evolving knowledge that tumor stroma plays an active role in cancer progression as it interacts with tumor and benign cells at different stages, from tumor initiation to invasion and metastasis [39, 40], explains our findings. We recommend recording the presence of keloid-like collagen stroma as a high grade of malignancy if it constitutes more than a third of the entire extracellular matrix of the tumor’s invasive margin. Future studies are needed to discover additional features of the collagen matrix that could more accurately predict patient treatment outcomes.

The strengths of this study are the homogeneous study population and a single geographic area where diagnosis and treatment were performed in the same hospital without selection bias. Other strengths were the comprehensive histological evaluation by specialized pathologists and the availability of detailed clinicopathological information. Limitations include the relatively small sample size compared to other studies in this area and the lack of detailed cancer treatment data. However, we adjusted multivariate models for clinical and demographic characteristics. In addition, because data on the histopattern of stromal collagen were not generally available to treating physicians, treatment decisions were not made based on the specific structural patterns of collagens in the desmoplastic reaction. This study also included patients from 5 to 10 years ago, and developments in colorectal cancer treatments during this period may have influenced prognosis. For these reasons, extensive multicenter studies are needed to explore trends further. Manual assessment of the relative amount of keloid-like collagen could have a relatively high variability of results, so in the future, digital assessment of colorectal cancer extracellular matrix proteins will significantly increase accuracy and reduce the variability of results.

## Conclusion

In summary, the results of this study indicate that keloid-like collagen stroma type B, when the relative amount of keloid-like collagen increases by more than 30% of the entire extracellular matrix in the tumor invasive front, is an independent prognostic factor in patients with TNM stage II or III colorectal cancer treated with surgery, with worse overall and recurrence-free survival rates. Determining the histophenotype of stromal keloid-like collagen will help stratify risk groups of patients and improve individual therapeutic strategies for colorectal cancer. Furthermore, these results suggest that stromal classification, in particular for stromal collagen histophenotype, may provide reliable prognostic features and improve TNM classification for colorectal cancer.

## Data Availability

The raw data supporting the conclusion of this article will be made available by the authors, without undue reservation.
